# Reply to Babich, “Using exosomes for universal vaccines”

**DOI:** 10.1128/iai.00207-25

**Published:** 2025-07-09

**Authors:** Saloni Bhimani, Mariola J. Ferraro

**Affiliations:** 1 Department of Microbiology and Cell Science, IFAS, University of Florida 3463, Gainesville, Florida, USA; University of California Davis, Davis, California, USA

## REPLY

Thank you for your generous commentary on our recent publication and your thoughtful consideration of how the exosome-binding peptide (EBP) described by Gurriaran-Rodriguez et al. (1) might intersect with our work on vesicle-based vaccination strategies.

We share your enthusiasm for the potential of host-derived extracellular vesicle-targeted antigen delivery, particularly in the context of mucosal immunity. However, we must note an important limitation in translating this directly into a plug-and-play vaccine strategy: in our studies, the immunogenic efficacy of small extracellular vesicles (sEVs) was closely tied to their generation in the context of infection. The protective effects we observed were not simply due to the presence of microbial antigens but also likely involved host-derived signals embedded within the vesicles that reflect the inflammatory environment and immune state of the producing cells.

In several studies (2–4), we demonstrated that small extracellular vesicles produced by *Salmonella*-infected macrophages elicit potent mucosal and systemic immune responses, including SIgA production and antigen-specific Th1 responses. Proteomic analyses confirmed that these vesicles carry both pathogen and host-derived antigens (e.g., OmpA, OmpD, and SopB) (4), but the precise host or bacterial proteins responsible for the observed immunogenicity remain unidentified. Compounding to the story, our lipidomics study (5) revealed significant changes in lipid composition of EVs during infection, which may alter vesicle uptake and immune signaling as well. These differences suggest that EVs formed under non-infectious or engineered conditions may not replicate the immune-stimulating potential of those formed *in vivo* during active infection, and it may possibly be even lipids that carry these immunogenic effects (5, 6).

This aligns with Xia and colleagues’ recent perspective article (7), which emphasizes that EV immunogenicity is context-dependent, influenced by origin cell type, activation state, surface markers, and EV corona content (e.g., CD47 and MHC molecules), as well as biophysical properties. They caution that engineered or synthetic EVs may require additional optimization to achieve consistent immunogenic profiles.

Regarding the EBP system, we agree that this discovery opens a new path for targeted vesicle cargo loading. However, while the ability to direct antigens like adhesins or pilins into EVs is conceptually interesting and promising, the immunological outcomes of such engineered vesicles are still unknown. As you noted, whether these vesicles need to co-deliver immunogenic cargo or possess a certain molecular “signature” to induce mucosal or T cell immunity remains an open question.

We believe the next phase of research should integrate these molecular targeting tools (like EBP) with understanding gained from infection-driven EV studies to build a functionally validated platform for vaccine design. Our most recent research work (2) tested sEV-based vaccines across genetically diverse *Salmonella* strains from wastewater and showed that immunity induced by infected-cell-derived vesicles could extend to circulating environmental strains—an encouraging sign of translational potential, but also a reminder of how context-sensitive these vesicles are.

We appreciate your forward-looking vision and fully agree that, given the urgent global health challenges posed by mucosal infections, for which there are few or no vaccines, we cannot afford to ignore this promising avenue of research. The EBP tool and our foundational studies provide a basis for further exploration, but we emphasize the need for rigorous validation of immunogenicity and functional protection when engineering vesicles outside of infection contexts.
